# Sources of Vitamin A in the Diets of Pre-School Children in the Avon Longitudinal Study of Parents and Children (ALSPAC)

**DOI:** 10.3390/nu5051609

**Published:** 2013-05-15

**Authors:** Victoria L. Cribb, Kate Northstone, David Hopkins, Pauline M. Emmett

**Affiliations:** 1 School of Social and Community Medicine, University of Bristol, Bristol, BS8 2BN, UK; E-Mails: vickycribb@hotmail.co.uk (V.L.C.); kate.northstone@bristol.ac.uk (K.N.); 2 Bristol Royal Hospital for Children, Paul O’Gorman Building, Upper Maudlin Street, Bristol, BS2 8BJ, UK; E-Mail: david.hopkins3@virgin.net

**Keywords:** vitamin A, carotene, retinol, core and non-core foods, pre-school children, ALSPAC, nutrient-dense foods, nutrient-poor foods

## Abstract

Vitamin A is essential for growth and development. We investigated whether high consumption of energy-dense nutrient-poor foods in the diets of pre-school children is detrimental to diet quality with respect to vitamin A. Data were collected from 755 children at 18-months and 3½-years, from the Avon Longitudinal Study of Parents and Children, using 3-day unweighed dietary records completed by parents in 1994 and 1996, respectively. Energy, carotene and retinol intakes were calculated. The quality of the diet declined from 18-months to 3½-years with respect to vitamin A. Preformed retinol intakes decreased by −54 μg/day on average (*p* = 0.003). Carotene intakes were similar at each age although there was a 23% increase in energy intake by 3½-years. Longitudinally those in the highest quartile of intake at 18-months were twice as likely to remain in the highest quartile at 3½-years for retinol (OR 2.21 (95% CI 1.48–3.28)) and carotene (OR 1.66 (95% CI 1.11–2.50)) than to change quartiles. Nutrient-rich core foods provided decreasing amounts of carotene and preformed retinol over time (both *p* < 0.001). Vegetables and milk contributed the highest proportion of carotene at both ages, but milk’s contribution decreased over time. Milk and liver were the largest sources of retinol. Nutrient-poor foods provided an increased proportion of energy (*p* < 0.001) with low proportions of both nutrients; however fat spreads made an important contribution. It is recommended that pre-school children should take vitamin supplements; only 19% at 18-months did this, falling to 11% at 3½-years. Care should be taken to choose nutrient-rich foods and avoid energy-dense, nutrient-poor foods when feeding pre-school children.

## 1. Introduction

Vitamin A is an essential nutrient and plays an important role in the diets of pre-school children. It can be obtained naturally in two forms; pre-formed as retinol and as pro-vitamin A (carotenoids) [1,2]. Vitamin A is important for optimal functioning of the immune system, vision, growth and bone development [3] and low intakes can reduce a person’s ability to fight infections [1]. Deficiency can increase the likelihood of a range of diseases and is the main cause of blindness in children; an estimated 250,000–500,000 lose their sight worldwide each year [4]. In adults, some studies have found a link between diets low in beta-carotene and some cancers [5], e.g., a higher intake of green and yellow vegetables associated with a decreased risk of lung cancer [6]. However, other studies have been unable to show this protective effect [7]. Vitamin A is also involved in vitamin D function, as these nutrients work in tandem through the same receptor sites [8]. There is some evidence that large intakes of retinol can adversely affect the action of vitamin D in the uptake of calcium in the bones [9] and reduce the protective effect of high vitamin D on colon cancer risk [10].

Pre-formed retinol is fat-soluble and well absorbed in the body and is found mainly in animal-based foods such as liver, butter, cheese, eggs, salmon, mackerel, herring and is added to most fat spreads [3]. The plant-based carotenoids (over 600 types) are found primarily in colourful fruits and vegetables such as carrots, sweet potato, green vegetables, tomatoes, oranges, cantaloupe melons and also in milk and butter and are added to fat spreads. However, only a few of the carotenoids have vitamin A activity and they are not as well absorbed as retinol [3].

In the UK, according to the National Diet and Nutrition Survey (NDNS 1992), intakes of vitamin A (assessed cross-sectionally) decreased with age from 1½ to 3½-years [11], but deficiency was not common. Supplementation for under fives, has been recommended by the World Health Organisation for more than 10-years [12], with supplements being distributed in numerous low and middle-income countries [13], however there are major criticisms of the effectiveness of supplementation. In the UK, it has been recommended since the 1990s that all children between 1 and 5 years of age be given supplements containing Vitamins A, C and D [14] with a revision of the recommendations for the Healthy Start (previously Welfare Food) scheme [15]. These children’s vitamin drops are free to mothers qualifying for Healthy Start vouchers and may be purchased by other mothers if they wish. Uptake is variable, as they are poorly advertised in health centres and are not always available and only 12% of 1½–3 year olds took supplements in the NDNS conducted in 2008–2010 [16].

Evidence suggests that establishing and maintaining healthy eating habits is important and habits formed in early life are likely to continue into adulthood [17]. Food preferences and food-related experiences during the first 2-years are associated with dietary variety in later childhood [18]. UK children’s diets, in general, are not meeting current food guidelines, with many consuming too much fat, sugar and salt [19], thus risking over-consumption of energy. Foods that are energy-dense and nutrient-poor have been classified in the Australian Guide to Healthy Eating as non-core foods; these include crisps, chips, cakes and biscuits. These foods are often referred to as “junk” foods [20,21] and countries such as the UK and US recommend a restricted intake of these non-core foods [22,23]. Foods that provide essential nutrients in a balanced way have been classified as “core” foods.

We aimed to examine the dietary sources of carotene and retinol in pre-school children in the UK, to determine whether deficiency of intake is likely in this developed country setting. We followed the same children at 18-months and 3½-years of age using a longitudinal birth cohort and assessed whether the consumption of energy-dense nutrient-poor foods is detrimental to the quality of the diet eaten by pre-school children. 

## 2. Materials and Methods

### 2.1. Subjects

Subjects were participating in the Avon Longitudinal Study of Parents and Children (ALSPAC), a longitudinal birth cohort study designed to investigate the health and development of children. ALSPAC has been described fully elsewhere [24,25]. Briefly, pregnant women were eligible to participate if they had an expected date of delivery between April 1991 and December 1992 and were resident in the former Avon Health Authority in South West England. A cohort of 14,541 pregnancies, resulting in 13,988 children alive at 12-months was established and a 10% random sample of mothers from the final 6-months of recruitment was invited to bring their child to a research clinic for in-depth assessments during their early years. Of the 1453 children who ever attended this part of ALSPAC 755 (52%) have provided dietary data at two pre-school ages and are included in this study. These children were biased toward having mothers with high education levels (A-levels or above) compared to mothers in the whole ALSPAC cohort (44.3% and 35.4%, respectively). Ethical approval for the study was obtained from the ALSPAC Law and Ethics Committee and the Local Research Ethic Committees.

### 2.2. Dietary Assessment

Dietary data were collected from the child’s main caregiver at two clinic visits; when the child was aged 18-months in 1994 and again aged 3½-years in 1996. The full methods have been described elsewhere [26,27]. In summary, prior to the clinic visit, caregivers were asked to record in a structured diary (using household measures) all food and drink consumed by the child over 3-days; 2 weekdays and 1 weekend day (self-selected and not necessarily consecutive), with a description of any leftovers. At the clinic they were interviewed by a nutrition fieldworker to clarify any anomalies. At 3½-years a short questionnaire asked about the use of vitamin supplements, types of fat spread normally used on bread and other details of foods commonly eaten, in order to aid coding. 

The completed diaries were coded using the computer program DIDO (Diet In, Data Out) developed by the MRC Human Nutrition Research Unit [28]. The portion sizes used were informed by descriptions in the food records with information from the second edition of the ‘Food Portion Sizes’ book [29] and from given weights of manufactured foods. The databank used for nutrient analysis included the 5th edition of McCance and Widdowson’s food tables [30] and supplements [31,32,33,34,35]. Additional current nutrient information was obtained from a national nutritional database and manufacturers’ information and covered all foods eaten. The nutrients for each food the child ate, with average energy, carotene and retinol content of each food group were calculated. The percentage contribution to these nutrients was calculated. Nutrient intakes from vitamin supplements were not included because details of their formulation were not recorded. Vitamin A requirements are generally expressed in terms of retinol equivalents to describe total vitamin A activity and in a normal balanced diet; 6 μg carotene is equivalent to 1 μg retinol [23]. The carotene available in the food tables is β-carotene equivalents and is the sum of the β-carotene and half the amount of α-carotene and α- and β-cryptoxanthins content of the food to provide a reasonable approximation of its vitamin A activity. The calculated nutrient intakes were similar to those from weighed food records collected in 1992 from NDNS, a nationally representative sample of similar age children [11,26,27]. An updated version of NDNS was carried out in 2008–2010 and children ate very similar foods to those eaten by the previous NDNS 1992 sample however fruit intake had increased considerably [16]. Intake of retinol equivalents from food sources only was 545 μg/day in these 1.5–3 year olds (*n* = 219).

### 2.3. Statistical Methods

In order to assess the quality of the diet in a meaningful way; foods were divided into core and non-core foods as defined by the Australian Guide to Healthy Eating [20]. Core foods are nutrient-rich and include: (1) bread, cereals, rice, pasta and noodles; (2) vegetables, legumes and potatoes (not chips); (3) fruit; (4) yoghurt, cheese, milk and (5) meat, fish, poultry, eggs and nuts. Non-core foods (listed in table below) tend to be energy-dense and nutrient-poor and include processed foods with added fat and/or sugar. Fat spreads are also classified as non-core although they are a good source of fat-soluble vitamins. Fats and oils for frying are included with fat spreads. Water and milk are classified as core beverages, all other drinks as non-core [21]. These other drinks, including sugar-sweetened beverages, contributed very little to vitamin A intake so have not been included. In order to facilitate direct comparisons between particular food groups consumed at the two different ages, the nutrient intakes from the food groups of interest were each divided by the respective average total energy intakes (MJ per day) to obtain relative nutrient intakes. Means and standard deviations for these energy-adjusted nutrients were calculated for each food group at each age. The changes in intake of the nutrients and changes in energy contribution between the two ages, overall and in the aggregated food groups, were assessed by paired *t*-test (mean difference with 95% confidence intervals (CI)). Total carotene and retinol intakes at both time-points were categorised into quartiles. The tracking of the nutrients from 18-months to 3½-years was assessed by using a multinomial logistic regression to estimate the odds ratio (OR, (95% CI)) for being in the same quartile (highest or lowest) for the nutrient at both ages compared to changing quartile. All statistical analyses were performed using SPSS for Windows, version 18 (SPSS Inc., Chicago, IL, USA). 

## 3. Results

### 3.1. Dietary Intakes

Retinol intakes decreased between the two ages from a mean of 425 μg/day to 371 μg/day (−54 μg, (CI −90, −18) *p* = 0.003). However carotene intakes were similar at each age although energy intake increased by 23% (Table 1). At 3½-years 24% of children did not meet the UK RNI recommendation (Table 1) for retinol equivalents [23]. Less than 2% were below LRNI. The US RDA recommendation is set at a lower level than UK RNI [36] so fewer children were below this. Children in the highest quartile of carotene intake at 18-months were 66% more likely to remain in the highest quartile at 3½-years (OR 1.66 (CI 1.11, 2.50)) than to change quartiles. There was a similar pattern in the lowest quartile (OR 1.51 (CI 1.01, 2.28)). For retinol intakes, children in the highest quartile at 18-months were more than twice as likely to remain in this quartile at 3½-years (OR 2.21 (CI 1.48, 3.28)) while those in the lowest quartile tracked slightly less well (OR 1.69 (CI 1.14, 2.51)). At 18 months, 19% consumed vitamin supplements decreasing to 11% at 3½-years. Children in the lowest quartile of intake of retinol equivalents were no more likely to take supplements than children in other quartiles at either age. 

**Table 1:** Mean (standard deviation) daily energy, carotene, retinol and retinol equivalent intakes in a cohort of children (*n* = 755) at 18 months and 3½-years and the proportion of children below target intakes. Change in intake over time, mean difference (95% confidence intervals) and significance by paired *t*-test.

	18-Months	3½-Years	Difference (95% CI) *p*-value
Energy (MJ)	4.61 (0.91)	5.67 (1.07)	
Carotene (μg)	1438 (1008)	1423 (1113)	−15 (82, −111) 0.770
Retinol (μg)	425 (413)	371 (311)	−54 (−18, −90) 0.003
Retinol equivalents (μg)	664 (443)	608 (372)	−56 (−17, −95) 0.005
Current UK recommendations ^1^			
UK LRNI ^a^ for retinol equivalents	200 μg/day	200 μg/day	
UK EAR ^b^ for retinol equivalents	300 μg/day	300 μg/day	
UK RNI ^c^ for retinol equivalents	400 μg/day	400 μg/day	
% below RNI recommendation	19%	24%	
% below LRNI	1%	2%	
Current US recommendations ^2^			
US RDA^d^ retinol equivalents	300 μg/day	300 μg/day	
% below recommendation	5%	10%	

^1^ [23]; ^2^ [36]; ^a^ Lower Reference Nutrient Intake (LRNI) is the amount of a nutrient that is enough for only the small number of people who have a low requirement (2.5%); ^b^ Estimated Average Requirement (EAR) is an estimate of the average requirement for a nutrient, approximately 50% of the population will need less and 50% of the population will need more; ^c^ Reference Nutrient Intake (RNI) is the amount of a nutrient that is enough to ensure that the needs of nearly all the population (97.5%) are being met; ^d^ Recommended Daily Allowance (RDA) is defined as “the average daily dietary intake level that is sufficient to meet the nutrient requirements of nearly all (approximately 98 percent) healthy individuals”.

### 3.2. Carotene and Retinol Intakes from Core and Non-Core Foods at Each Age

Table 2 shows the energy-adjusted carotene and retinol intakes from core food items and the percentage contribution from food sources for energy and both nutrients. Table 3 presents similar results for non-core foods. The core foods provided 250 μg/MJ/day carotene at 18-months decreasing to 202 μg/MJ/day at 3½-years (−48 μg/MJ (CI −30, −67), *p* < 0.001) while the non-core foods increased from 18 to 22 μg/MJ/day respectively (4 μg/MJ (CI 2, 6), *p* < 0.001). At 18 months core foods provided 72 μg retinol/MJ/day but this decreased to 46 μg/MJ/day by 3 years (−25 μg/MJ (CI −18, −32), *p* < 0.001), with the contribution from non-core foods increasing from 14 to 17 μg/MJ/day (4 μg/MJ (CI 3, 5), *p* < 0.001). Foods such as breast and formula milk and baby foods have not been included in the tables because they were not eaten at both ages; at 18-months 7% of retinol and 6% of carotene came from these foods. In contrast to their nutrient contributions core foods provided 63 and 54% (difference −9% (CI −8, −10), *p* < 0.001) of the energy while non-core foods provided 31 and 42% at 18-months and 3½-years respectively (difference 11% (CI 10, 12), *p* < 0.001).

Of the core foods, vegetables and the milk group contributed the highest proportion of carotene to the diet, but intakes decreased between the two ages, particularly from milk. Intakes of retinol from core foods were primarily provided by milk and cheese, and meat, particularly liver/offal, provided a considerable proportion. However, the contribution from the meat group decreased markedly over time (Table 2).

**Table 2:** Energy-adjusted carotene and retinol (mean (standard deviation) μg/MJ/day) and percentage contribution to energy of core food items in a cohort of children (*n* = 755) at 18-months and 3½-years. Food group intakes of carotene and retinol compared between 18 months and 3½-years by paired *t*-test.

Core foods	18-Months					3½-Years				
	Contribution to energy (%)	Carotene μg/MJ	%a	Retinol μg/MJ	%b	Contribution to energy (%)	Carotene μg/MJ	%a	Retinol μg/MJ	%b
*Bread, cereals, rice & pasta*										
Bread	7.1	0.0 (0.1)	0.0	0.0 (0.2)	0.1	9.2	0.1 (0.3)	0.0	0.1 (0.6)	0.2
Breakfast cereals	5.6	0.1 (0.6)	0.0	0.2 (1.4)	0.2	5.6	0.1 (0.5)	0.0	0.2 (1.2)	0.3
Pasta, rice and savouries	2.7	9.4 (21.8)	3.1	0.6 (1.9)	0.7	3.4	8.2 (19.3)	3.2	1.1 (2.7)	1.7
Group total	*15.3*	*9.5 (21.8)*	*3.1*	*0.9 (2.4)*	*1.1*	*18.2* *	*8.4 (19.3)*	*3.3*	*1.5 (3.0)* *	*2.2*
*Vegetables & legumes*										
Vegetables	1.5	204.3 (216.0)	65.4	0.0 (0.1)	0.0	1.3	158.6 (201.0)	63.7	0.0 (0.2)	0.0
Potatoes	2.1	0.6 (1.3)	0.2	0.7 (1.4)	0.7	1.7	0.6 (1.2)	0.2	0.6 (1.3)	0.9
Vegetable dishes	0.3	2.3 (14.9)	0.7	0.3 (1.7)	0.3	0.3	3.1 (19.7)	1.3	0.4 (2.0)	0.6
Legumes	0.1	0.1 (0.8)	0.0	0.0 (0.0)	0.0	0.1	0.2 (2.2)	0.1	0.0 (0.0)	0.0
Group total	4.0*	*207.2 (215.6)* *	*66.3*	*1.0 (2.2)*	*1.1*	*3.3*	*162.5 (201.5)*	*65.3*	*1.0 (2.4)*	*1.6*
*Fruit*										
Fruit	4.4	4.8 (8.9)	1.5	0.0 (0.0)	0.0	3.4	5.0 (16.1)	2.0	0.0 (0.0)	0.0
Fruit juice	1.5	1.0 (2.4)	0.3	0.0 (0.0)	0.0	1.8	1.6 (6.5)	0.7	0.0 (0.0)	0.0
Group total	5.9 *	*5.8 (9.3)*	*1.8*	*0.0 (0.0)*	*0.0*	*5.2*	*6.6 (17.3)*	*2.7*	*0.0 (0.0)*	*0.0*
*Yoghurt, cheese & milk*										
Milk	25.1	14.8 (10.9)	5.0	42.1 (23.0)	44.8	15.6	9.8 (8.1)	3.9	25.4 (17.5)	39.8
Yoghurt	4.0	0.5 (1.0)	0.2	4.0 (4.7)	4.3	3.2	0.4 (0.8)	0.2	3.5 (4.4)	5.5
Cheese	2.3	2.9 (4.1)	0.9	4.5 (5.9)	4.8	2.6	3.3 (4.4)	1.3	5.0 (6.4)	7.7
Group total	31.4 *	*18.1 (11.4)* *	*6.1*	*50.5 (23.5)* *	*54.0*	*21.4*	*13.5 (9.0)*	*5.4*	*34.0 (18.6)*	*53.0*
*Meat, fish, poultry & eggs*										
Meat	2.7	7.8 (26.2)	2.6	15.9 (82.9)	19.2	2.4	8.2 (26.7)	3.5	6.7 (42.2)	9.5
Fish	0.5	0.1 (0.7)	0.0	0.2 (0.9)	0.2	0.5	0.1 (0.8)	0.0	0.2 (0.8)	0.3
Poultry	1.2	0.9 (7.0)	0.3	0.2 (0.4)	0.1	1.6	2.0 (13.9)	0.7	0.2 (0.6)	0.3
Eggs & egg dishes	1.2	0.7 (5.4)	0.2	2.9 (6.2)	3.2	1.1	0.6 (4.6)	0.2	2.8 (5.8)	4.2
Group total	*5.7*	*9.5 (27.8)*	*3.2*	*19.1 (82.9)* *	*22.6*	*5.6*	*10.9 (30.7)*	*4.4*	*10.0 (42.3)*	*14.4*
*Core foods total*	62.8 *	*250.1 (216.6)* *	*80.5*	*71.5 (86.7)* *	*78.8*	*53.8*	*201.8 (204.9)*	*81.1*	*46.4 (46.4)*	*71.2*

%a, Percentage contribution to carotene; %b, Percentage contribution to retinol; * Significantly higher intake by paired *t*-test, *p* < 0.001.

Contributions to both nutrients from non-core foods were quite low. Fat spreads provided the highest proportion of both carotene and retinol at each age. The contribution to retinol intake of the cakes, biscuits and snacks group doubled over time from 4.4 to 8% with a larger increase in its contribution to energy from 18 to 25% (7% (CI 8, 6), *p* < 0.001) (Table 3).

**Table 3:** Energy-adjusted carotene and retinol (mean (standard deviation) μg/MJ/day) and percentage contribution to energy of non-core food items in a cohort of children (*n* = 755) at 18-months and 3½-years. Food group intakes of carotene and retinol compared between 18 months and 3½-years by paired *t*-test.

Non-core Foods	18-Months					3½-Years				
	Contribution to energy (%)	Carotene μg/MJ	%a	Retinol μg/MJ	%b	Contribution to energy (%)	Carotene μg/MJ	%a	Retinol μg/MJ	%b
*Miscellaneous*										
Puddings & ice-creams	3.3	1.1 (2.6)	0.4	1.8 (3.2)	1.9	4.3	2.0 (4.0)	0.8	2.2 (3.7)	3.4
Buns, cakes & pastries	2.8	1.4 (2.8)	0.5	1.7 (3.3)	1.7	4.3	1.9 (3.6)	0.8	2.0 (3.8)	3.1
Sweet biscuits	4.9	0.3 (1.1)	0.1	0.4 (1.5)	0.4	5.7	0.3 (1.5)	0.1	0.4 (1.7)	0.6
Savoury biscuits	0.7	0.1 (0.2)	0.0	0.1 (0.5)	0.1	0.6	0.0 (0.1)	0.0	0.1 (0.3)	0.2
Confectionery	4.8	0.6 (1.1)	0.2	0.3 (0.4)	0.3	7.1	1.2 (2.2)	0.5	0.4 (0.6)	0.6
Crisps	2.5	1.4 (3.4)	0.6	0.0 (0.0)	0.0	4.3	1.4 (3.3)	0.6	0.0 (0.0)	0.0
Group total	*19.1*	*5.0 (5.1)*	*1.7*	*4.3 (4.8)*	*4.4*	*26.3* *	*6.9 (6.8)* *	*2.8*	*5.2*	*8.0*
*Processed meat, fish & poultry*										
Processed meats	0.8	0.0 (0.2)	0.0	0.0 (0.2)	0.0	1.0	0.0 (0.1)	0.0	0.0 (0.0)	0.0
Coated chicken	0.3	0.0 (0.0)	0.0	0.0 (0.0)	0.0	0.9	0.0 (0.0)	0.0	0.0 (0.0)	0.0
Burgers & kebabs	0.2	0.0 (0.0)	0.0	0.0 (0.0)	0.0	0.2	0.0 (0.1)	0.0	0.0 (0.1)	0.0
Sausages	1.1	0.0 (0.0)	0.0	0.0 (0.2)	0.0	1.4	0.0 (0.0)	0.0	0.0 (0.3)	0.0
Meat pies	0.8	0.4 (3.7)	0.2	0.1 (0.5)	0.2	0.9	0.5 (3.8)	0.2	0.3 (1.2)	0.5
Coated & fried fish	1.0	0.0 (0.0)	0.0	0.0 (0.1)	0.0	1.3	0.0 (0.0)	0.0	0.0 (0.0)	0.0
Group total	*4.3*	*0.4 (3.7)*	*0.2*	*0.2 (0.6)*	*0.2*	*5.8* *	*0.5 (3.8)*	*0.2*	*0.3*	*0.5*
*Vegetables*										
Fried/roast potatoes	2.9	0.0 (0.0)	0.0	0.0 (0.1)	0.0	4.3	0.0 (0.0)	0.0	0.0 (0.1)	0.0
Baked beans	1.2	2.3 (3.8)	0.8	0.0 (0.0)	0.0	0.9	1.9 (3.1)	0.8	0.0 (0.0)	0.0
Group total	*4.1*	*2.3 (3.8)*	*0.8*	*0.0 (0.1)*	*0.0*	*5.3* *	*1.9 (3.1)*	*0.8*	*0.0*	*0.0*
*Spreads, soup & sauces*										
Fat Spreads	3.3	5.4 (4.3)	1.8	8.6 (6.3)	9.2	4.3	6.6 (4.8)	2.6	11.3 (7.7)	17.7
Soup	0.3	3.1 (21.7)	1.1	0.3 (1.1)	0.2	0.3	3.6 (17.8)	1.3	0.3 (1.2)	0.5
Milk-based sauces	0.2	0.2 (0.8)	0.1	0.3 (1.2)	0.3	0.1	0.1 (0.7)	0.0	0.2 (1.0)	0.3
Tomato-based sauces	0.1	0.5 (1.9)	0.2	0.0 (0.0)	0.0	0.2	1.1 (4.5)	0.4	0.0 (0.1)	0.0
Other sauces	0.5	0.7 (3.3)	0.2	0.0 (0.2)	0.0	0.6	0.8 (3.1)	0.4	0.0 (0.2)	0.0
Group total	*4.3*	*9.9 (22.4)*	*3.3*	*9.2 (6.5)*	*9.7*	*5.5* *	*12.2 (19.2)*	*4.8*	*11.9 (7.9)* *	*18.4*
Non-core foods total	*31.8*	*17.6 (23.7)*	*6.0*	*13.7 (8.3)*	*14.3*	*42.8* *	*21.5(20.4)* *	*8.6*	*17.4 (9.4)* *	*26.9*

%a, Percentage contribution to carotene; %b, percentage contribution to retinol; * significantly higher intake by paired *t*-test, *p* < 0.001.

The change between core and non-core foods in the overall balance of contribution to energy and the nutrients was mainly due to the relative decline in milk consumption and increase in consumption of biscuits, cakes, confectionery and crisps.

### 3.3. The Contribution of Particular Foods

Within the vegetable group cooked carrots provided the most carotene (750 and 647 μg/day at respective ages), with other cooked vegetables (36 and 33 μg/day) and green leafy vegetables (36 and 28 μg/day) the next highest, while raw carrots increased in prominence (26 to 92 μg/day). Whole milk provided considerable amounts of carotene (65 μg/day average intake) compared to other types of milk (3 μg/day from semi-skimmed at 18-months). Beef and beef dishes contributed more carotene at both ages compared with other meat sources, at 3½-years 42 μg compared with 5 μg from lamb and lamb dishes. This is probably due to vegetables in stews and other dishes. The highest food contributor to retinol was whole milk at 3½-years it provided 126 μg/day, with semi-skimmed the next best provider, 16 μg/day. Liver and liver dishes provided the majority of the retinol within the meat group (73 and 41 μg/day at respective ages), with other meats providing negligible amounts. Butter (12 and 20 μg/day) and full-fat polyunsaturated margarine (15 and 26 μg/day) contributed the most retinol within fat spreads. 

## 4. Discussion

This study found evidence that the quality of children’s diet declined from age 18-months to 3½-years with respect to both carotene and retinol. At 3½-years the children drank less milk and obtained more energy from non-core foods including biscuits, cakes, and confectionery, than at 18-months. Milk, particularly whole milk, made an important contribution to both nutrients but non-core foods made only a limited contribution. There was also a decline in the use of vitamin supplements. Children in this study were eating a more energy-dense, nutrient-poor diet at 3½-years than at 18-months. This is of concern since this type of diet has been linked to the rising prevalence of childhood obesity [37].

In the current sample, up to 24% of children were not meeting the RNI for retinol equivalents and less than 20% were taking supplements despite the recommendation that all children of this age in UK should be given them [14]. Very few were below the LRNI confirming that vitamin A deficiency is unlikely to be common in these children. In findings from the NDNS 2008-10, slightly more of the participants (9%) were below the LRNI for retinol equivalents, dropping to 8% when dietary supplements were included because only 12% took any type of vitamin supplement [16]. In both the NDNS and this study vitamin supplement use was too low to guarantee adequate intake for all children particularly as those with low dietary intake in our study were no more likely to take supplements than those with higher intake. A substantial proportion of the carotene component of vitamin A came from vegetables thus adding weight to the healthy eating guidelines which promote vegetable intake. For pre-formed retinol, full-fat dairy products were important and butter and fat spreads, which are fortified with both nutrients, contributed more in the older children. 

Absorption of fat-soluble vitamins is enhanced by dietary fat. Diets of young children should therefore contain some fat and foods such as whole milk, yoghurt and cheese play an important role in a child’s diet [38].The contributions from milk to both carotene and retinol declined between the two ages. This was due to both a reduction in the amount and a change in the type consumed. Whole milk contains 21 μg of carotene/100 g compared with 9 μg/100 g from semi-skimmed and none from skimmed milk. Likewise, whole milk contains considerably more retinol (52 μg/100 g) than other types of milk (21 and 1 μg/100 g for semi-skimmed and skimmed respectively) [30]. This study endorses the recommendations that whole milk is a suitable drink from the age of 1-year while semi-skimmed milk should only be introduced if the child is a good eater and growing well from the age of 2-years, and skimmed milk should not be used for the under fives, primarily because it does not contain fat or fat-soluble vitamins [14,38]. In some countries, such as USA, it is recommended that lower fat milks are fortified with vitamin A and D to at least the levels contained in whole milk, however this is not always mandatory so it is left with milk producers to decide whether to do it. It is usually made clear that the food should be labeled if fortification has occurred.

The percentage contribution to retinol from meat decreased substantially between the ages. This is explained by the reduced consumption of liver and may be due to increased levels of pickiness (fussy eating) that tend to occur at this age, resulting in a less varied diet [39]. Fussy eaters tend to have limited intakes of fruit and vegetables [40], and higher intakes of unhealthy snack foods characterised by their high sugar and fat content.

There was evidence of tracking of both carotene and retinol intakes from 18-months to 3½-years. Those children consuming the highest level of both nutrients at one age were twice as likely to be consuming the highest level at the next age. Thus it is likely that introducing nutrient-rich diets early will continue to be beneficial. We could find no other studies that examined tracking of these nutrients in pre-school children. 

There is some concern over toxicity of pre-formed vitamin A. In children it is suggested that consumption levels of 20 times the RNI could be toxic. It is very unlikely that children even with high dietary intakes could reach toxic levels [41].

Important strengths of this study are its relatively large sample size and the comprehensive dietary data available on the same children at two time-points during early childhood; to our knowledge no other study can match this. With our sample size of 755 children we were able to detect, for example, a mean change of 5.6 μg/MJ/day (standard deviation of the difference 55 μg/MJ/day) in carotene over time, based on 80% power and alpha set to 0.05. However, the study was conducted in one geographical area and thus results may not be characteristic of the whole UK population. Although the ALSPAC cohort was reasonably representative of the UK at recruitment [25] in this study we only included children from a sub-sample with complete dietary data at two ages so there was a bias towards higher education levels in the mothers. Intakes of nutrients were estimated from 3-day un-weighed food records completed by untrained caregivers and standard food tables were used to provide information about the likely nutrient content of the foods. These methods can lead to further biases especially since only a limited number of days of intake were assessed and these may not be representative of diet over time. However these biases would apply equally at each age thus the differences we found between the ages are likely to be less affected. We have presented energy-adjusted food group data to enable direct comparison of food group nutrient intakes between the ages. For comparison with other data absolute intakes may be obtained by multiplying by the appropriate energy intake provided in Table 1. Our dietary data were collected in 1994 and 1996 and have been shown to be comparable with NDNS 1992 which increases our confidence in it. However, it is possible that our results may not reflect current dietary intake. A recent update of the NDNS shows very similar overall intakes of vitamin A with if anything a decline in the adequacy of the diet; 9% of children aged 1.5-3-years received less than the LRNI for vitamin A [16] compared to 2% in this study and the level of supplement use had not increased. Taking these biases together suggests that our study is more likely to under- than over-estimate current dietary problems.

## 5. Conclusion

In conclusion, the importance of the quality of the diet in achieving an adequate intake of vitamin A in these young children has been underlined. Changing the type and amount of milk consumed reduced the amount of carotene and retinol obtained and the use of fortified fat spreads enhanced intake. Care must be taken to provide nutrient-rich core foods and avoid energy-dense nutrient-poor non-core foods when feeding pre-school children. 

## Implications

High intakes of commonly consumed energy-dense nutrient-poor foods in preschool children are detrimental to their intake of vitamin A and the quality of their diet tends to decline with age. More attention should be paid to feeding nutrient-rich foods during early childhood.
